# *CsATG101* Delays Growth and Accelerates Senescence Response to Low Nitrogen Stress in *Arabidopsis thaliana*

**DOI:** 10.3389/fpls.2022.880095

**Published:** 2022-05-10

**Authors:** Wei Huang, Danni Ma, Xulei Hao, Jia Li, Li Xia, E. Zhang, Pu Wang, Mingle Wang, Fei Guo, Yu Wang, Dejiang Ni, Hua Zhao

**Affiliations:** ^1^Key Laboratory of Horticultural Plant Biology of Ministry of Education, Huazhong Agricultural University, Wuhan, China; ^2^College of Horticulture and Forestry Sciences, Huazhong Agricultural University, Wuhan, China

**Keywords:** *CsATG101*, autophagy, nitrogen deficiency, tea plant, senescence

## Abstract

For tea plants, nitrogen (N) is a foundational element and large quantities of N are required during periods of roundly vigorous growth. However, the fluctuation of N in the tea garden could not always meet the dynamic demand of the tea plants. Autophagy, an intracellular degradation process for materials recycling in eukaryotes, plays an important role in nutrient remobilization upon stressful conditions and leaf senescence. Studies have proven that numerous autophagy-related genes (*ATGs*) are involved in N utilization efficiency in *Arabidopsis thaliana* and other species. Here, we identified an *ATG* gene, *CsATG101*, and characterized the potential functions in response to N in *A. thaliana*. The expression patterns of *CsATG101* in four categories of aging gradient leaves among 24 tea cultivars indicated that autophagy mainly occurred in mature leaves at a relatively high level. Further, the *in planta* heterologous expression of *CsATG101* in *A. thaliana* was employed to investigate the response of *CsATG101* to low N stress. The results illustrated a delayed transition from vegetative to reproductive growth under normal N conditions, while premature senescence under N deficient conditions in transgenic plants vs. the wild type. The expression profiles of 12 *AtATGs* confirmed the autophagy process, especially in mature leaves of transgenic plants. Also, the relatively high expression levels for *AtAAP1*, *AtLHT1*, *AtGLN1;1*, and *AtNIA1* in mature leaves illustrated that the mature leaves act as the source leaves in transgenic plants. Altogether, the findings demonstrated that *CsATG101* is a candidate gene for improving annual fresh tea leaves yield under both deficient and sufficient N conditions *via* the autophagy process.

## Introduction

Nitrogen (N) is an essential nutrient in plants for the synthesis of amino acids, proteins, and many other N-containing currency forms during the whole life. The tea plant [*Camellia sinensis* (L.) O. Kuntze] is a perennial evergreen beverage plant with tender leaves for harvest, and large quantities of N are required due to the roundly plucking and pruning (Chen et al., 2012; Zhang et al., 2020). In tea plantations, to achieve a top-quality and considerable yield, the base fertilizer and topdressing are two essential batches of fertilizer. Usually, the base fertilizer is applied following the cessation of growth of the aboveground tea plants, while the topdressing is a fast-released fertilizer applied seasonally. Thus, the optimum time for topdressing is of great importance for tea plants (Watanabe, 1995), and it should match the seasonal growth rhythm (Zhang et al., 2020). However, the fluctuating N availability in soil is not sufficient to meet the real-time requirement of the tea plants. Under such adverse environments of N deficiency, intracellular recycling systems play critical roles in nutrients delivery from earlier sources to sinks (Marmagne et al., 2020).

Plants have employed sophisticated mechanisms to survive the limited nutrient supply by recycling intracellular constituents (Thomas, 2013). The ubiquitin-26S-proteasome system is a well-studied recycling system in which proteins are decorated by ubiquitin molecules and degraded by 26S proteasome (Su et al., 2020). However, this process is insufficient for recycling some individually damaged proteins and bulk degraded proteins during leaf senescence (Chen et al., 2019a). Autophagy, an evolutionarily conserved vacuolar pathway in plants, is another intracellular nutrient recycling system to maintain the homeostasis within a cell or plants under such conditions of senescence and N limitation (Himelblau and Amasino, 2001; Li F. et al., 2015). The core process of autophagy is mainly mediated by four distinctly functional groups, (i) the Atg1 protein kinase complex, mainly functioning in autophagy initiation; (ii) ATG9/2/18 transmembrane complex, delivering membranes for autophagosome formation; (iii) the PI3K (phosphatidylinositol 3-kinase) complex, mediating vesicle nucleation; and (iv) the ubiquitin-like ATG8-PE (phosphatidylethanolamine) conjugation pathway and ATG12-ATG5 conjugation pathway, participating in the elongation and enclosure steps during autophagosome formation (Avila-Ospina et al., 2014; Masclaux-Daubresse et al., 2017).

During the process of autophagy, several autophagy-related genes (*ATGs*) are involved (Tsukada and Ohsumi, 1993; Kim et al., 2012), among which, many *ATGs* have been functionally characterized in N utilization across a series of plant species (Avin-Wittenberg et al., 2018; Chen et al., 2019b). In *A. thaliana*, the ^15^N pulse-chase experiments showed that *atg5*, *atg9*, and *atg18a* were strongly affected in N remobilization from source leaves to seeds (Guiboileau et al., 2012). Later, Di Berardino et al. (2018) found that *atg5* is concerned with the protein storage accumulation in seeds under both ample and limited nitrate conditions. In rice (*Oryza sativa*), the mutant *Osatg7-1* presented early visible leaf senescence with a high N concentration remaining in senescent leaves, demonstrating that *Osatg7-1* contributes to N recycling from senescent to young leaves (Wada et al., 2015). Recently, studies have demonstrated that overexpression of *OsATG8a* (Yu et al., 2019), *OsATG8b* (Fan et al., 2020; Zhen et al., 2021), and *OsATG8c* (Zhen et al., 2019a) increase the nitrogen use efficiency (NUE) and/or nitrogen remobilization efficiency in rice. In apple (*Malus Domestica*), *MdATG8i* (Wang et al., 2016), *MdATG9* (Huo et al., 2020), and *MdATG18a* (Sun et al., 2018) have been proven to improve tolerance to N starvation. These studies mainly employed the mutants or homologous expression of the genes in the autologous plants for function analysis. In some plants without a well-established transformation system, heterologous expression of genes in model plants was reported, such as a soybean gene *GmATG8c* (*Glycine max*) conferring tolerance to N deficiency in *A. thaliana* (Xia et al., 2012), a foxtail millet gene *SiATG8a* (*Setaria italica*) improving N starvation tolerability in *A. thaliana* and rice (Li W. et al., 2015; Li et al., 2016), and a rice gene *OsATG8b* conferring tolerance to N starvation and, meanwhile, increasing NUE in *A. thaliana* (Zhen et al., 2019b). *In Camellia sinensis*, autophagy is also essential for nutrient recycling (Chen et al., 2020). It was demonstrated that the expression levels of *CsATG5*, *CsATG9*, *CsATG12*, and *CsATG18* in leaves were strongly influenced by the removal of flower buds (Fan et al., 2019). All these research illustrated that the physiological roles of *ATGs* involved in N utilization have been widely studied in the processes of plant autophagy (Qi et al., 2021).

In most eukaryotes (budding yeast not included), *ATG101* is one of the members composed of the Atg1 protein kinase complex, which is assumed to be involved in the initiation of autophagy (Hosokawa et al., 2009; Mercer et al., 2009; Noda and Fujioka, 2015). Several studies related to *ATG101* have focused on the HORMA (Hop 1, Rev 7, and Mad 2) fold structure (Hegedűs et al., 2014; Suzuki et al., 2015; Kim et al., 2018), as well as the regulation in autophagy initiation (Qi et al., 2015; Guo et al., 2019). However, the function of *ATG101* associated with N indices is scarcely understood.

In tea plants, *CsATG101* is a single copy (Huang et al., 2020). It was reported that the expression level of *CsATG101* changed with abiotic stress, such as drought and cold (Wang et al., 2021). However, whether it contributes to N utilization efficiency has not been discussed in the literature. Here, we investigated the potential functions of *CsATG101* in response to different N conditions. We first examined the expression patterns of *CsATG101* in four categories of leaves with gradient maturity among 24 tea cultivars. Further, we overexpressed *CsATG101* in Col-0 ecotype *A. thaliana* (WT) and aimed to characterize its function in response to the two contrast N levels under both hydroponic solution and soil conditions. We also investigated the gene expression patterns affected by *CsATG101* in *A. thaliana*. It was shown that overexpression of *CsATG101* in *A. thaliana* showed better growth of rosette leaves and delayed the developmental transition from vegetative to reproductive growth under normal N conditions, whereas it accelerated the senescence of mature leaves of *A. thaliana* under N deficient condition. Therefore, our results demonstrated that *CsATG101* is assumed to improve the growth of tender tea shoots under both N-sufficient and deficient conditions.

## Materials and Methods

### Tea Plant Materials

**FIGURE 1:**
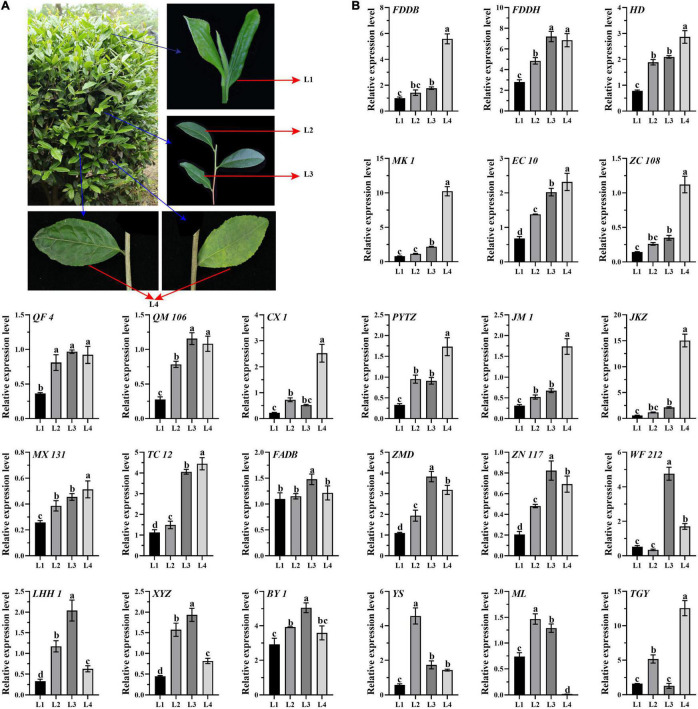
Expression analysis of *CsATG101* in the four categories of leaves of 24 tea cultivars. **(A)** Representative tea plant branches for sampling. L1, one bud with two developing leaves; L2, the recently developed leaves attached to the green stems L3, the mature leaves attached to the red stem; L2 and L3 developed in the same year as L1; and L4, aged leaves developed last year. **(B)** Expression patterns of *CsATG101* in the four categories of leaves of 24 tea cultivars. *CsGAPDH* was used as the internal control, and the sample of L1 for FDDB was the control sample. Three technical replicates were performed. Data are means ± *SD*. Significant differences are indicated by letters (ANOVA; *P* ≤ 0.05).

To reveal the genes expression patterns in a list of gradient mature leaves, leaf samples were collected, including L1 (one bud with two developing leaves), L2 (the recently developed leaves attached to the green stems), L3, (the mature leaves attached to the red stems), L2 and L3 developed in the same year as L1, and L4 (aged leaves developed last year) (Figure 1A). A total of 24 10-year-old tea cultivars with different processing suitability of green tea, oolong tea, black tea, and white tea were selected (Supplementary Table 1), including Fuding Dabaicha (FDDB), Fuding Dahaocha (FDDH), Fuan Dabaicha (FADB), Tie Guanyin (TGY), Huangdan (HD), Mingke 1 (MK 1), Zimudan (ZMD), Echa 10 (EC 10), Zhongcha 108 (ZC 108), Zhenong 117 (ZN 117), Qianfu 4 (QF 4), Qianmei 601 (QM 601), Wufeng 212 (WF 212), Chuxiang 1 (CX 1), Lenghouhun (LHH 1), Pingyang Tezaocha (PYTZ), Jiaming 1 (JM 1), Xianyuzao (XYZ), Jiukengzao (JKZ), Maolv (ML), Mingxuan 131 (MX 131), Yingshuang (YS), Taicha 12 (TC 12), and Baiye 1 (BY 1). All the 24 cultivars were grown on the tea plantation of Huazhong Agricultural University (Wuhan, Hubei, China). Samples were collected on September 28th, 2017, frozen immediately in liquid nitrogen, and stored at −80^°^C for RNA extraction.

### *CsATG101* Cloning and Analyses of Amino Acid Sequences

The coding sequence (CDS) of *CsATG101* was first amplified with non-restriction enzyme primers (listed in Supplementary Table 2) from the cDNA of Fuding Dabaicha and cloned into the pTOPO vector (Aidlab, Beijing, China) following the manufacturer’s instructions. Further, it was confirmed by sequencing and alignment against the tea plant genome in the CSS_ChrLev_20200506 version at Tea Plant Information Archive (TPIA).^1^ And the gene homologous were BLASTed using the protein sequence of CsATG101 in Ensembl Plants.^2^ The amino acid sequence alignment was performed using DNAMAN V6 (Lynnon Biosoft, Foster City, CA, United States).

### Generation of Transgenic *Arabidopsis thaliana* Plants

The CDS of *CsATG101* was first excised with *Xba* l/*Xho* l from pTOPO, and then, ligated into pBin35SRed (Zhang et al., 2015). Subsequently, the constructs were introduced into *Agrobacterium tumefaciens* stain GV3101. Then, an *A. thaliana* wild type (WT, Col-0) transformation was performed using floral dipping (Clough and Bent, 1998). Finally, two independently homozygous T3 lines, namely line 1 and line 2, at a relatively high expression level of *CsATG101* (Supplementary Figure 1) were selected for the following experiments. The primers used in vector construction are shown in Supplementary Table 2.

### *Arabidopsis thaliana* Culture Conditions and Nitrogen Regimes

The harvest of T3 seeds and the method for hydroponic culture were conducted as described previously (Huang et al., 2020). Three weeks after transferring from the petri dish, plants were divided into two groups and treated with LN (Low N, 0.125 mM NH_4_NO_3_) and NN (normal N, 2.5 mM NH_4_NO_3_), respectively. Two weeks later, tissues of roots, mature and young leaves of rosette were collected and stored at −80^°^C after freezing instantly in liquid nitrogen for gene expression analysis. For the determination of biomass and N accumulation, the plants were separated into roots, rosettes, young leaves, mature leaves, and aerial parts excluding rosette (APER). Also, for the measurement of amino acid-N, the inflorescences, roots, mature leaves, and young leaves from rosette were harvested. Each sample consisted of four individual plants at least.

Under a hydroponic culture, in the pod setting stage, it is difficult to support the plants vertically and to reduce seed pods’ crack. To determine the effect of *CsATG101* on seeds production, both the transgenic plants (T3) and WT were grown in soil in a growth chamber (22^°^C/18^°^C ± 2^°^C, 8 h light/16 h dark). First, seeds were put at 4^°^C for stratification for 2 days and then sown in pots with a mixture of nutritious soil substrate and vermiculite (70%:30%, w:w, 100 g/pot^–1^) (22^°^C ± 2^°^C) for seed germination without light. Then, the 4-week-old seedlings were grouped into two groups of LN and NN treatments. Then, in the next 4 weeks, a supply of 28 mg N kg^–1^ and 560 mg N kg^–1^ was conducted for LN and NN, respectively, which were averagely applied with the NH_4_NO_3_ as the exogenous N four times, i.e., 0.7 mg N pot^–1^ for LN and 14 mg N pot^–1^ for NN each time. During the following week, the 9-week-old plants were at the transition stage from vegetative to reproductive growth, and a comparison of vegetative growth and N indices was investigated between transgenic plants and WT at the two contrast N levels. Finally, another 6 weeks later, the seeds and stubble leaves (without roots) were sampled to analyze the reproductive growth between transgenic plants and WT at the end of the seed maturity.

### RNA Extraction and Gene Expression Analysis

The total RNA was extracted using the Quick RNA Isolation Kit following a recommended protocol (Huayueyang, Beijing, China). Then, the RNA was treated with genomic DNA (gDNA) Eraser to remove DNA contaminants (Aidlab, Beijing, China). The complementary DNA (cDNA) first-strand synthesis was done using TRUEscript RT Kit (Aidlab, Beijing, China) with 1 μg RNA. The resulting cDNA samples were diluted at 1:10 and a quantitative real-time polymerase chain reaction (qRT-PCR) was performed using an ABI StepOnePlus Real-Time PCR System (Applied Biosystems) with SYBR Green qPCR Mix (Aidlab, Beijing, China) according to the manufacturer’s instructions. Fold changes in gene expression were determined by comparing the C_*T*_ values using the 2^–Δ^ ^Δ^ *^CT^* method (Livak and Schmittgen, 2001) with the control genes of *CsGAPDH* (*Camellia sinensis*) and *AtGAPDH* (*A. thaliana*). Three independently grown sets were performed as biological replicates, and the qRT-PCR of each biological replicate was detected three times at least. The primers used for qRT-PCR were listed in Supplementary Table 2.

### Determination of Nitrogen and Amino Acid-Nitrogen in *Arabidopsis thaliana*

Samples of roots, young leaves, and mature leaves from rosettes, and APER were kept in a dry oven at 120^°^C for 10 min, followed by incubation at 75^°^C for 5 days to a constant weight. To measure the N concentration, the dried samples were firstly ground into a fine powder, followed by digestion with H_2_SO_4_-H_2_O_2_. The concentration of N was determined using a flow injection analysis instrument (FIAstar 5000 analyzer; FOSS, Hilleroed, Denmark). The N accumulation in each part was calculated with the biomass and N concentration. To measure the amount of amino acid-N, 0.15 g fresh samples of inflorescences, roots, mature leaves, and young leaves from rosette were weighed, and then, measured using the Ninhydrin Colorimetric Analysis method (Rosen, 1957). For each determination, three independent replicates were carried out at least.

### Statistical Analysis

The One-way ANOVA Duncan at *P* ≤ 0.05 was used to detect significant differences among tissues for tea plants, transgenic *A. thaliana* plants, and WT using the SPSS 21.0 software (IBM). All the figures were performed for windows using the software of GraphPad Prism 8.0.1 (San Diego, California, United States) and Adobe Illustrator CC2019 (ADOBE, United States).

## Results

### Molecular Cloning and Sequence Analysis of *CsATG101*

We identified a homologous sequence of *AtATG101* from Fuding dabaicha and named it *CsATG101*. The CDS length of this gene is 657 bp, encoding a 218 amino acid-deduced protein. Protein alignment between CsATG101 and other orthologous ATG101 proteins presented a similarity of 54.13% for *Oryza sativa* and 58.64% for *Zea mays*, 75.80% for *A. thaliana* and the other woody plant species, with the highest of 88.99% with *Actinidia chinensis*. As no canonically recognizable domain exists despite a strictly conserved WF finger among ATG101 proteins (Li et al., 2014; Noda and Mizushima, 2016), here, we also confirmed a WF finger in CsATG101 protein (Supplementary Figure 2). Among the 657 bp coding sequences, relative to Fuding dabaicha, eight-position changes with synonymous variants occurred among the sequenced cultivars of Shuchazao, Tieguanyin, and Longjing 43, and a change in position 221 from A to G leads to a missense variant from lysine to arginine in Suchazao and Longjing 43, which is out of the WF finger. However, whether there is a differential function caused by this allelic variation remains unknown.

### The Expression Level of *CsATG101* Is Higher in Mature Leaves Than in Developing Leaves of Tea Plants

To evaluate the expression patterns of *CsATG101* among tissues in the tea plant, we first employed the data from TPIA among four tissues, including the apical bud, young leaf, mature leaf, and old leaf. As shown in Supplementary Figure 3, the *CsATG101* was detected among all four tissues, with an increased tendency from the apical bud, and young leaves to mature leaves. However, a decrease was also captured between matured leaves and old leaves.

In tea plants, nutrients availability in the maturity-gradient leaves changes dynamically. To investigate the expression pattern of *CsATG101* in detail, qRT-PCR was conducted for four categories of leaves with gradient maturity among 24 tea cultivars. As shown in Figure 1B, it presented a remarkable increase in expression level in L2 vs. L1 in eighteen out of 24 cultivars, whereas no noticeable difference was observed for the six other cultivars, including FDDB, MK 1, ZC 108, JKZ, FADB, and WF 212. Intriguingly, the expression level of *CsATG101* was significantly higher in both L3 and L4 in contrast to L1 for nineteen out of 24 cultivars. For five other cultivars, a remarkably higher expression level was also observed either in L3 or in L4, exactly, FADB, BY 1, and ML for L3 vs. L1, CX 1, and TGY for L4 vs. L1. Nevertheless, in the cultivars of YS and ML, a remarkable decrease in transcript level was found from L2 to L4. However, seven cultivars, FADB, ZMD, ZN117, WF212, LHH, XYZ, and BY 1, showed an obvious lower level in L4 in contrast to L3. Interestingly, in TGY, an higher level was captured in L3 compared with both L2 and L4. In total, the higher expression level of *CsATG101* in the mature leaves in comparison to developing leaves indicated that an increased probability of autophagy occurs with tea leaves maturity, though it varies between aging gradient leaves, as well between cultivars.

### *CsATG101* Delays the Bolting Under NN and Accelerated the Senescence Under LN in *Arabidopsis thaliana*

**FIGURE 2:**
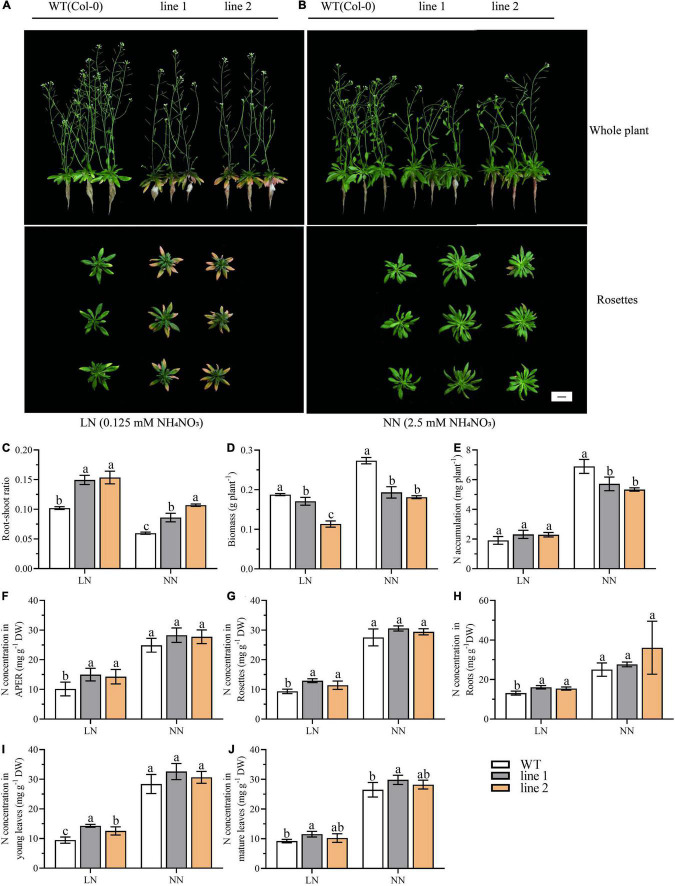
The *CsATG101* overexpression and wild type *A. thaliana* in response to N levels. Plants were hydroponic cultured for 3 weeks under NN and then under LN **(A)** and NN **(B)** for 2 weeks. **(C)** Root-shoot ratio (Root biomass/shoot biomass). **(D)** Biomass of whole plant (*n* ≥ 5). **(E)** Total N accumulation at whole plant level (*n* ≥ 5). The N concentration in APER **(F)**, Rosettes **(G)**, Roots **(H)**, Young leaves **(I)**, and Mature leaves **(J)** (*n* ≥ 5). Three biological replicates were performed. Data are means ± *SD*. Significant differences are indicated by letters (ANOVA; *P* ≤ 0.05). WT, wild type (Col-0); line 1 and line 2, two transgenic lines; APER, Aerial part excluding rosette. LN, low nitrogen, 0.25 mM N (0.125 mM NH_4_NO_3_); NN, normal nitrogen, 5 mM N (2.5 mM NH_4_NO_3_). Scale bar = 2 cm.

Our earlier data also found that the expression level of *CsATG101* changed with N levels (Supplementary Figure 4). To evaluate the potential function of *CsATG101* related to N use, we investigated and compared the growth between transgenic *A. thaliana* plants and WT growing under both LN (0.125 mM NH_4_NO_3_) and NN (2.5 mM NH_4_NO_3_) conditions. As shown in Supplementary Figure 5, cultured at NN level for 3 weeks, the transgenic plants showed delayed transition from vegetative to reproductive stage compared with WT. Two weeks after LN treatment, the rosette leaves of transgenic plants turned yellow with an exacerbation from young to mature rosette leaves, whereas the pale-yellow color on the margin of some rosette leaves of WT was observed (Figure 2A). Under the condition at normal N level, only a few mature leaves of transgenic plants became slightly yellow on the margins. Additionally, the diameter of rosette leaves was larger in transgenic plants than in WT, while the height of transgenic plants was relatively shorter compared with WT (Figure 2B). Together, these results illustrated that the heterologous *CsATG101* in *A. thaliana* accelerated the senescence under N deficiency while it enhanced the rosette leaves growth under NN conditions.

### *CsATG101* Decreases the Biomass and the Nitrogen Accumulation in *Arabidopsis thaliana* Irrespective of Nitrogen Conditions

To investigate how *CsATG101* affects *A. thaliana* growth under different N conditions, the biomass and N accumulation of transgenic plants and WT were analyzed. As seen in Figure 2C, a striking increase in root-shoot ratio was displayed in transgenic plants in contrast to WT under both LN and NN conditions, as well, an increase was observed under LN compared with NN, suggesting a stronger adaptive performance to low N in transgenic plants than WT. By contrast, the biomass was significantly decreased in transgenic plants (60.4%–91.0% and 66.2%–70.7% of WT under LN and NN, respectively) vs. WT (Figure 2D). The biomass allocation in APER significantly decreased by 85.4%–86.8% of WT under LN and 52.6%–69.2% under NN compared with control plants (Supplementary Figure 6G). The decrease was mainly caused by the biomass of APER instead of rosettes or roots (Supplementary Figures 6A–C).

Under LN, the N concentration was significantly higher in transgenic plants than that in the control plants, with 141%–147.9%, 122.3%–138.3%, and 117.6%–122.3% of that in WT for APER, rosettes, and roots, respectively (Figures 2F–H). In young leaves and mature leaves, an increased tendency of N concentration could be observed in transgenic *A. thaliana* plants compared with that in WT plants (Figures 2I,J). However, N accumulation in transgenic plants was not significantly different from that of WT, as well as the accumulated N in APER (Figure 2E and Supplementary Figure 6H). Supplementary Figures 6D–F showed that the slightly elevated N accumulation in rosettes and roots contributed little to the whole plant. By contrast, under NN, an increase in the N concentration between transgenic plants vs. WT was observed for APER, rosettes, and roots, though the differences were not significant (Figures 2F–H). However, the N accumulation at the whole plant level and the accumulation allocated into APER showed significant decreases of 77.4%–82.9% and 51.8%–67.5%, respectively, in transgenic plants compared with WT (Figure 2E and Supplementary Figure 6H). Obviously, in Supplementary Figures 6D–F, the results demonstrated the dramatical decrease in APER dominated the whole plant N accumulation and the allocation between transgenic plants and WT, despite the increase of N accumulation in rosettes. Especially, in the aerial part, a higher percentage of N accumulation in young leaves and mature leaves was found in overexpression plants than in WT plants (Supplementary Figure 6I).

**FIGURE 3:**
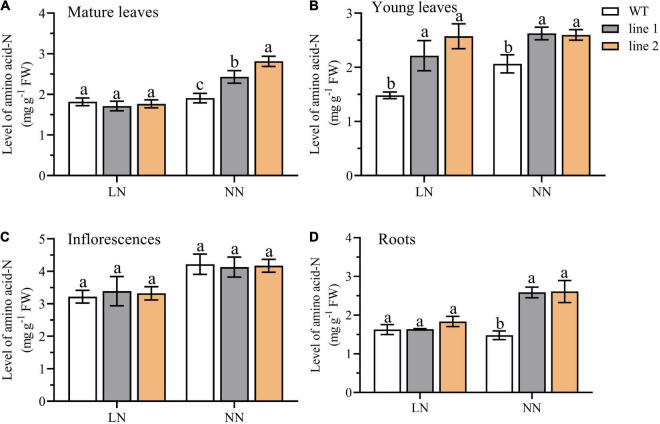
A comparison of the amino acid-N level in mature leaves **(A)** and young leaves **(B)** of rosette, inflorescences **(C)**, and roots **(D)** between the two transgenic *Arabidopsis* lines and wild type. Plants were hydroponically cultured for 3 weeks under NN, and then under LN and NN for 2 weeks (*n* ≥ 5). Three biological replicates were performed. Data are means ± *SD*. Significant differences are indicated by letters (ANOVA; *P* ≤ 0.05). WT, wild type (Col-0), line 1 and line 2, two transgenic lines. LN, low nitrogen, 0.25 mM N (0.125 mM NH_4_NO_3_); NN, normal nitrogen, 5 mM N (2.5 mM NH_4_NO_3_).

Amino acid-N indicates the status of available N within the plant. To further evaluate the potential free N availability for plants, we examined the level of N derived from the free amino acids pool (amino acid-N) in four parts of plants, including roots, inflorescences, mature leaves, and young leaves of the rosette. As depicted in Figure 3, under LN, the amino acid-N level in the young leaves of rosette was significantly higher (149.5%–173.7% of WT) in transgenic plants than in the control plants (Figure 3B), while the levels of amino acid-N in roots, inflorescences, and mature leaves of rosette were not significantly different from those in WT (Figures 3A,C,D). However, under NN conditions, a remarkable enhancement was observed in transgenic plants vs. WT among the parts of mature leaves of the rosette, young leaves of rosette, and roots (Figures 3A,B,D). While no differences were detected for inflorescences (Figure 3C).

### Expression Patterns of Genes Involved in Nitrogen Uptake, Transport and Metabolism, and Autophagy in *Arabidopsis thaliana* Affected by *CsATG101*

**FIGURE 4:**
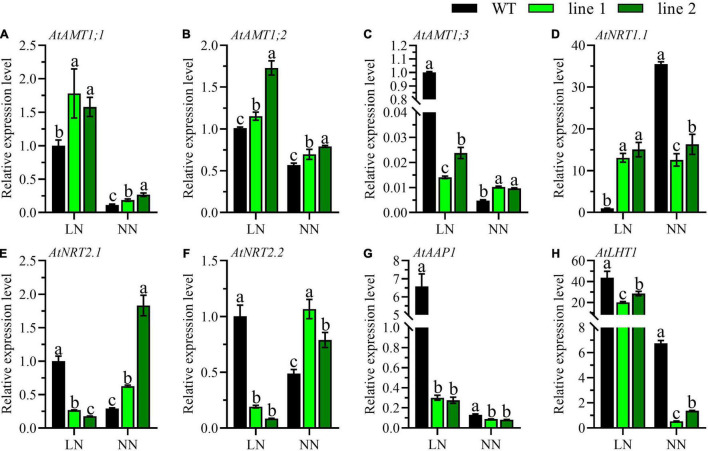
Comparable expression analysis of genes associated with N uptake and assimilation in the roots of two transgenic *Arabidopsis* lines and wild type. **(A–C)** Genes related with ammonium-N acquisition, **(D–F)** genes related with nitrate-N uptake, and **(G,H)** genes related with amino-N metabolism. Plants were hydroponically cultured for 3 weeks under NN, and then under LN and NN for 2 weeks (*n* ≥ 3). *AMT*, Ammonium transporter; *NRT*, Nitrate transporter; *AAP*, Amino acid permease; *LHT*, Lysine histidine transporter. *AtGAPDH* was used as the internal control. The expression level in young leaves of WT under LN was set as “1” for *AtAAP1* and *AtLHT1*, while for the rest genes, the expression level in roots of WT under LN was set as “1.” Three technical replicates were performed. Data are means ± *SD*. Significant differences are indicated by letters (ANOVA; *P* ≤ 0.05). WT, wild type (Col-0), line 1 and line 2, two transgenic lines. LN, low nitrogen, 0.25 mM N (0.125 mM NH_4_NO_3_); NN, normal nitrogen, 5 mM N (2.5 mM NH_4_NO_3_).

To reveal the influence caused by *CsATG101*, the expression patterns of fourteen genes involved in N uptake, transport, metabolism, and twelve genes in the autophagy process were determined. Ammonium transporters, nitrate transporters, and some amino acid transporters have been reported to be involved in N acquisition or metabolism in *A. thaliana* roots (Masclaux-Daubresse et al., 2010). Here, the expression patterns of eight genes, three *AMTs* for ammonium acquisition, three *NRTs* for nitrate uptake, and two genes for organic N metabolism, were analyzed in the roots of transgenic plants and WT. Under LN, the expression profiles of *AtAMT1;3*, *AtNRT2.1*, and *AtNRT2.2* were significantly downregulated in transgenic plants compared with WT (Figures 4C,E,F). In contrast, the genes, *AtAMT1;1*, *AtAMT1;2*, and *AtNRT1.1* showed the opposite expression patterns, and the transcript level of *AtNRT1.1* was marginally higher by a 15-fold change in transgenic plants in contrast to WT (Figures 4A,B,D). By contrast, under NN, a dramatical downregulation was shown for *AtNRT1.1* in transgenic plants vs. WT (Figure 4D). However, the expression levels of *AtAMT1;1*, *AtAMT1;2*, *AtAMT1;3*, *AtNRT2.1*, and *AtNRT2.2* were unanimously noticeable higher in transgenic plants than in WT (Figures 4A–C,E,F). Intriguingly, the expression patterns of *AtAAP1* and *AtLHT1* were significantly decreased in transgenic plants with a comparison of WT irrespective of N conditions (Figures 4G,H).

**FIGURE 5:**
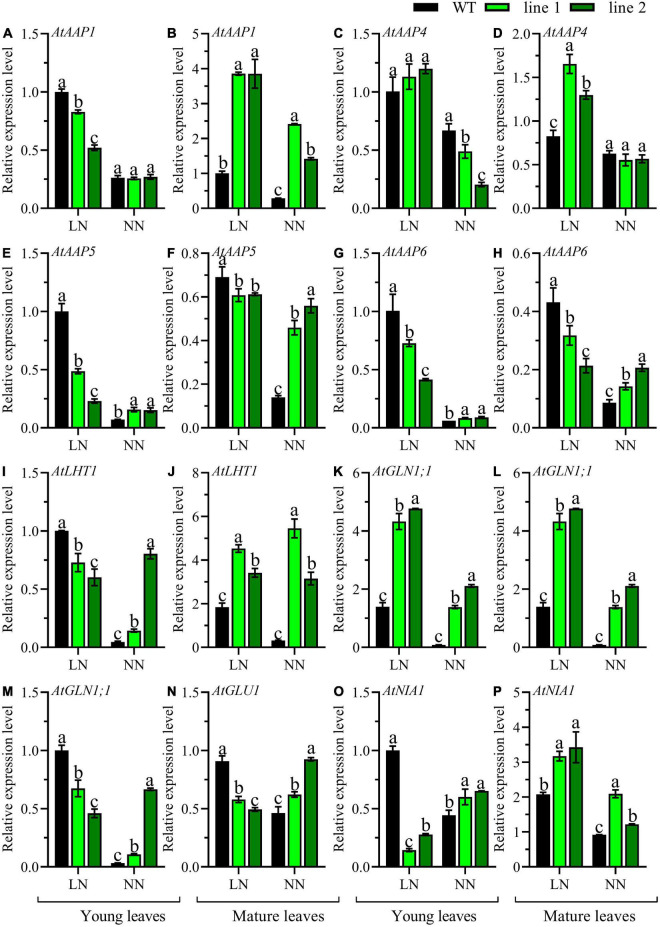
Comparable expression analysis of genes associated with N assimilation in the mature leaves and young leaves between two transgenic *Arabidopsis* lines and wild type. Plants were hydroponically cultured for 3 weeks under NN, and then under LN and NN for 2 weeks (*n* ≥ 3). *AAP*, Amino acid permease; *LHT*, Lysine histidine transporter; *GLN*, Gln synthetase; *GLU*, Gln 2-oxoglutarate aminotransferase; *NIA*, nitrate reductase. **(A,C,E,G,I,K,M,O)** Genes expressed in young leaves. **(B,D,F,H,J,L,N,P)** Genes expressed in mature leaves. *AtGAPDH* was used as the internal control, and the expression level in young leaves of WT under LN was set as “1” for each gene. Three technical replicates were performed. Data are means ± SD. Significant differences are indicated by letters (ANOVA; *P* ≤ 0.05). WT, wild type (Col-0), line 1 and line 2, two transgenic lines. LN, low nitrogen, 0.25 mM N (0.125 mM NH_4_NO_3_); NN, normal nitrogen, 5 mM N (2.5 mM NH_4_NO_3_).

The comparison of the expression patterns of eight genes involved in N metabolism, including five amino acid transporters (*AtAAP1*, *AtAAP4*, *AtAAP5*, *AtAAP6*, and *AtLHT1*) and three genes that participated in N assimilation (*AtGLN1;1*, *AtGLU1*, and *AtNIA1*) in the mature leaves and young leaves between transgenic plants and WT were conducted. Under LN, an upregulation of *AtAAP1*, *AtAAP4*, *AtLHT1*, *AtGLN1;1*, and *AtNIA1* (Figures 5B,D,J,L,P), and a downregulation in *AtAAP5*, *AtAAP6*, and *AtGLU1* (Figures 5F,H,N) was observed in mature leaves of transgenic plants when compared with WT. However, in young leaves, except for *AtAAP4* (Figure 5C), a significant downregulation was detected in transgenic plants vs. WT for the rest seven genes (Figures 5A,E,G,I,K,M,O). While under NN, a remarkable upregulation was observed in mature leaves of transgenic plants for all the genes except for *AtAAP4* maintained constantly among all the lines (Figures 5B,D,F,H,J,L,N,P). However, in young leaves, most of the tested genes displayed upregulation in transgenic plants (Figures 5E,G,I,K,O), and the expression level of *AtAAP4* was downregulated (Figure 5C), while no significant differences were observed for *AtAAP1* and *AtGLU1* (Figures 5A,M). Overall, the expression of genes responsible for N metabolism in transgenic plants displayed an upregulation in rosette both young and mature leaves under NN while a down-regulation in young leaves under LN except *AtAAP4*.

**FIGURE 6:**
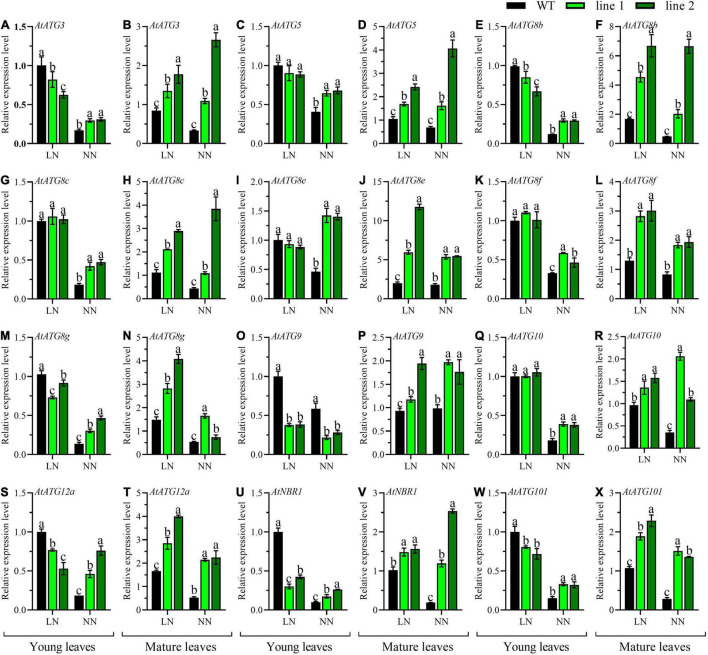
Comparable expression analysis of genes associated with autophagy in the young leaves and mature leaves between two transgenic *Arabidopsis* lines and wild type. Plants were hydroponically cultured for 3 weeks under NN and then under LN and NN for 2 weeks (*n* ≥ 3). *ATG*, autophagy related gene; *NBR1*, neighbor of BRCA1. **(A,C,E,G,I,K,M,O,Q,S,U,W)** Genes expressed in young leaves. **(B,D,F,H,J,L,N,P,R,T,V,X)** Genes expressed in mature leaves. *AtGAPDH* was used as the internal control, and the expression level in young leaves of WT under LN was set as “1” for each gene. Three technical replicates were performed. Data are means ± *SD*. Significant differences are indicated by letters (ANOVA; *P* ≤ 0.05). WT, wild type (Col-0), line 1 and line 2, two transgenic lines. LN, low nitrogen, 0.25 mM N (0.125 mM NH_4_NO_3_); NN, normal nitrogen, 5 mM N (2.5 mM NH_4_NO_3_).

Additionally, the expression patterns of 12 *AtATGs* in rosette were comparably investigated between transgenic plants and WT. As shown in Figures 6B,D,F,H,J,L,N,P,R,T,V,X, the expression levels for all the tested *AtATGs* in mature leaves of transgenic rosette were significantly higher vs. WT under both LN and NN. However, the differences in expression profiles in young leaves depended on N conditions. Under LN, the expression levels were not significantly different in five *AtATGs* (*AtATG5*, *AtATG8c*, *AtATG8e*, *AtATG8f*, and *AtATG10*, Figures 6C,G,I,K,Q) among plants, whereas seven *AtATGs* were significantly downregulated in transgenic plants compared with WT (Figures 6A,E,M,O,S,U,W). Under NN, 11 *AtATGs* were significantly upregulated for transgenic plants vs. WT (Figures 6A,C,E,G,I,K,M,Q,S,U,W), while *AtATG9* was downregulated in transgenic plants in contrast to that of the control plants (Figure 6O). Together, these results demonstrated that strengthened autophagy occurred in mature rosette leaves of transgenic plants in comparison with WT independent of N conditions; whereas in young rosette leaves, a similar tendency was still applicable under normal conditions, and a weakened autophagy might explain the difference between transgenic plants vs. WT under LN.

### *CsATG101* Increases the Nitrogen Allocation in Seeds Under Both LN and NN in *Arabidopsis thaliana*

**FIGURE 7:**
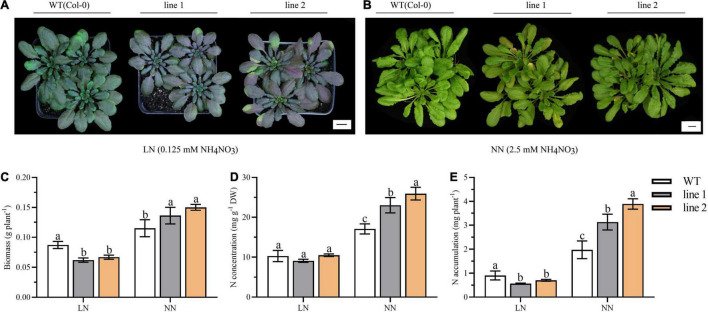
Biomass, N accumulation and growth performance in soil for 9-week-old transgenic and wild type *Arabidopsis theliana*. Plants grown for 9 weeks under LN **(A)** or NN **(B)** conditions. Biomass **(C)**, N concentration **(D)** and N accumulation **(E)** of desiccated plants harvested at vegetative phase (9 weeks; *n* ≥ 5). Three biological replicates were performed. Data are means ± *SD*. Significant differences are indicated by letters (ANOVA; *P* ≤ 0.05). WT, wild type (Col-0), line 1 and line 2, two transgenic lines. LN, low nitrogen, 0.25 mM N (0.125 mM NH_4_NO_3_); NN, normal nitrogen, 5 mM N (2.5 mM NH_4_NO_3_).

Autophagy contributes to the N remobilization from vegetative to reproductive organs (Havé et al., 2017). Thus, a comparison of transgenic plants and WT was investigated for the growth performance and N allocation under soil conditions. At the vegetative stage, both the transgenic plants and WT displayed purple under LN, which is particularly obvious in transgenic plants (Figure 7A). While under NN, all the plants kept green with slight yellowish in transgenic plants (Figure 7B).

We further analyzed the biomass and N accumulation at the vegetative stage. As shown in Figures 7C–E, under LN, the biomass and the N accumulation of transgenic plants was significantly reduced by 23.3%–29.0% and 22.4%–37.7% relative to WT, respectively. By contrast, under NN, for transgenic plants, the biomass varied between 0.1363 g/plant and 0.1501 g/plant, N concentration ranged from 23.0293–25.9471 mg/g, and N accumulation was (3.1303–3.8935) mg N/plant; all of them were significantly increased compared with those in WT plants.

**FIGURE 8:**
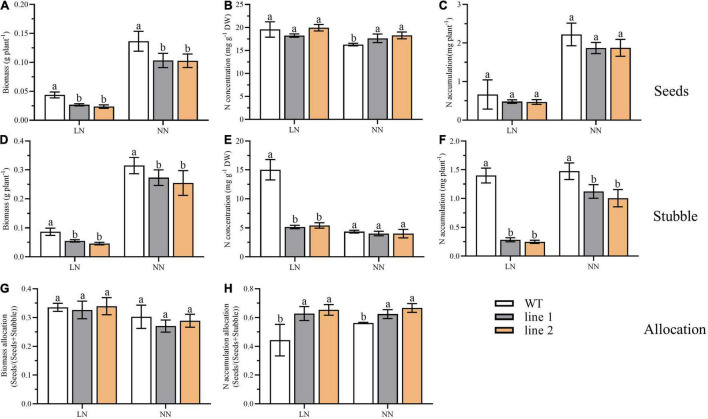
Biomass, N accumulation and allocation in soil at reproductive phase of two transgenic *Arabidopsis* lines and wild type. Biomass **(A)**, N concentration **(B)** and N accumulation **(C)** of desiccated seeds (*n* ≥ 5). Biomass **(D)**, N concentration **(E)** and N accumulation **(F)** of desiccated stubble (*n* ≥ 5). **(G)** The biomass allocation in seeds (seeds biomass/biomass of seeds and stubble) (*n* ≥ 5). **(H)** The N allocation in seeds (seeds N accumulation/N accumulation of seeds and stubble) (*n* ≥ 5). Three biological replicates were performed. Data are means ± *SD*. Significant differences are indicated by letters (ANOVA; *P* ≤ 0.05). WT, wild type (Col-0), line 1 and line 2, two transgenic lines. LN, low nitrogen, 0.25 mM N (0.125 mM NH_4_NO_3_); NN, normal nitrogen, 5 mM N (2.5 mM NH_4_NO_3_).

At the end of the reproductive stage, we sampled the plants into two separated parts, seeds and stubble (no roots included), and analyzed the biomass and N accumulation. As shown in Figure 8, under LN, the seeds yield, the biomass of stubble, N concentration, and N accumulation in the stubble of transgenic plants were significantly decreased by 53.9%–61.1%, 52.8%–63.6%, 34.3%–36.1%, and 17.7%–20.4% of those of WT, respectively (Figures 8A,D,E,F). Nevertheless, no obvious differences were detected in N concentration and N accumulation in seeds between transgenic plants and WT (Figures 8B,C). Under NN, as the seeds yield decreased and the N concentration increased, no significant difference was found in N accumulation branched into seeds between transgenic plants and WT (Figures 8A–C). The significantly decreased biomass and N accumulation in the stubble of transgenic plants were found compared with WT (Figures 8D,F). Although, in transgenic plants, the biomass allocation of seeds [seeds/(seeds + stubble)] was not significantly different (Figure 8G), the partition of N accumulation into seeds showed an obvious increase by 41.6%–47.3% at the LN level and 10.9%–18.5% at NN level (Figure 8H), respectively, in comparison with WT.

## Discussion

In plants, autophagy plays a pivotal role in nutrients recycling, especially for N (Guiboileau et al., 2013; Pottier et al., 2014; Havé et al., 2017). Previous studies have shown that many *ATGs* are involved in N utilization, especially under deficient conditions (Masclaux-Daubresse and Chardon, 2011; Islam et al., 2016; Wang et al., 2017). In the present study, *CsATG101*, a member of the initiation complex in autophagy, was cloned from Fuding Dabaicha. Analysis of the proteins indicates a WF finger in CsATG101 and a high similarity ranging from 54.13% to 88.99% was captured compared with several other species. Further, the expression patterns of *CsATG101* in four different ages of 24 tea cultivars were investigated. The globally increased expression level from L1 to L3 and/or L4 confirmed the previous conclusion, senescence stimulates autophagy for nutrient recycling in aging leaves while a basal level of autophagy occurs in the developing leaves (Hanaoka et al., 2002; Avila-Ospina et al., 2014; Woo et al., 2018). The difference in expression levels between L1 and L2 was not significant in six cultivars, suggesting both L1 and L2 were at a basal level of autophagy since they are younger parts. Further, in nine cultivars (FADB, ZMD, ZN117, WF212, CX 1, XYZ, BY1, YS, and ML), the expression levels were significantly lower in L4 compared with L3 or L2, which could be proven by the TPIA data in the tissue of old leaves to mature leaves as we presented in Supplementary Figure 3. This reduction is probably caused by the decline in L4, approaching senescence. However, an exception, TGY, showed instantly increased transcript level in L2 and it might be due to a huge N requirement in L1, suggesting L2 acting as the source leaf and L1 as the N sink leaf, as our previous study identified that the sources and sinks alternate in the developed leaves of tea plants (Zhang et al., 2020).

Heterologous expression of an exogenous gene in the model plant is a common way to investigate the function of genes (Chen et al., 2019b; Zhen et al., 2019b). In this study, heterologous expression of *CsATG101* in *A. thaliana* was used to investigate its potential function in response to N status. The transition from vegetative to the reproductive stage was delayed in the transgenic plants in comparison with WT under NN while accelerated the leaves senescence under LN, suggesting that *CsATG101* improves the vegetative growth under ample N conditions, while it is involved in nutrient recycling under N deficient condition. Consistent with the above findings, the biomass from both hydroponic and soil cultures confirmed our results (Supplementary Figures 6A–C and Figure 7C).

The uptake and assimilation of ammonium-N are tightly regulated by the expression of AMTs, *AtAMT1;1*, *AtAMT1;2*, and *AtAMT1;3* accounts for more than 90% of ammonium-N uptake (Tegeder and Masclaux-Daubresse, 2018). Here, we found a significantly elevated expression levels of *AtAMT1;1* and *AtAMT1;2* in transgenic plants vs. WT (Figures 4A,B), indicating the transgenic plants exhibited a higher ability in acquiring ammonium. Compared with the transcript level of *AtAMT1;3* of WT at LN, transgenic plants under both N levels and WT with NN showed extremely lower levels (Figure 4C), suggesting *AtAMT1;3* had been compensated by other *AMTs* as demonstrated in earlier reports (Duan et al., 2018). Nitrate is another important inorganic N and high-affinity and low-affinity nitrate transport systems are available in plants (Vidal et al., 2020). In this study, we investigated the expression patterns of two high-affinity nitrate transporters (*AtNRT2.1* and *AtNRT2.2*) and one dual-affinity nitrate transporter (*AtNRT1.1*). We found a distinct result that, compared with WT, the expression level of high-affinity nitrate transporters of *AtNRT2.1* and *AtNRT2.2* were significantly lower under LN, but elevated under NN in transgenic plants (Figures 4E,F), while *AtNRT1.1* showed a completely contrary profile (Figure 4D). The *CsATG101*-mediating contrast expression patterns under LN and NN were perhaps regulated by *AtNRT1.1*, a known nitrate transceptor (Undurraga et al., 2017; Vidal et al., 2020). In our study, NH_4_NO_3_ was the only N resource in the nutrient solution, thus, the *AMTs* and *NRTs* are the main transporters involved in N absorption from solution. Here, the expression patterns of *AtAMTs* and *AtNRTs* were not in uniform between transgenic plants and WT, indicating the differences of N uptake might lead to the differential N accumulation in plants as we have presented above.

Amino acid transporters play important roles in N circulation within plants as amino acids are the main components of N currency (Tegeder and Masclaux-Daubresse, 2018). *AtAAP1* functions in amino acid acquisition in root and subsequent translocation from roots to the shoot, as well contributing to seed development (Lee et al., 2007). *AtLHT1* plays roles in amino acid uptake in root and import into mesophyll cells (Hirner et al., 2006). In contrast to WT, *AtAAP1* and *AtLHT1* of transgenic plants displayed a notably lower level in roots especially under LN, implying there was a relatively less ability in N assimilation in the transgenic plants as no amino acid-N is available in solution (Figures 4G,H). Conversely, they expressed strikingly higher in the mature leaves of transgenic plants compared with WT under both LN and NN (Figures 5B,J), indicating a high possibility of amino acids into sink such as shoots and developing leaves under both LN and NN conditions.

The *AtAAP4*, mainly expressed in source leaves, stem and flowers (Fischer et al., 1995), was relatively higher expressed in transgenic plants under LN condition in comparison with WT. Under LN or NN, limited variation was indicated between mature leaves and young leaves of WT, but a slightly higher level in mature leaves relative to young leaves in transgenic plants indicated the mature leaves served as source leaves (Figures 5C,D). *AtAAP5* was reported to express in all tissues (Fischer et al., 1995). In this study, relative to WT, the contrast patterns of a decrease level under LN and an increase level under NN in transgenic plants illustrated that the transgenic plants improved the amino acids availability under NN (Figures 5E,F). *AtAAP6* is a transporter responsible for uptake of amino acids from xylem (Okumoto et al., 2002) and its expression patterns depend on N conditions. Exactly, under LN, the expression level of *AtAAP6* was higher in young leaves than mature leaves with a downregulated expression in transgenic plants vs. WT, while under NN, it was highly expressed in mature leaves and showed upregulated expression in transgenic plants compared with WT (Figures 5G,H), indicating the transgenic plants showed an efficient utilization of the amino acids from mature leaves under NN.

Three genes, *AtGLN1;1, AtGLU1*, and *AtNIA1* are involved in the N assimilation. *AtGLN1;1* presented a relative higher expression level in mature leaves of transgenic plants compared with WT under both LN and NN (Figure 5L), indicating that the *CsATG101* enhanced the N recycling from mature leaves to sink leaves, and the result is consistent with a previous literature demonstrating the role in nutrient transport during senescence (Li et al., 2006). *AtGLU1*, responsible for reassimilation of the ammonium released from photorespiration in leaves (Coschigano et al., 1998), was downregulated in transgenic plants in contrast to WT under LN (Figures 5M,N), indicating that a leak of ammonium might have caused by *CsATG101* under N deficiency. *AtNIA1*, a gene encoding nitrate reductase 1 and predominantly active in leaves (Olas and Wahl, 2019), was significantly higher expressed in mature leaves of transgenic plants relative to WT under both LN and NN (Figure 5P), indicating that *CsATG101* might contribute to the enhancement of nitrate utilization from mature leaves.

Additionally, the expression patterns of 12 *AtATGs* from differently functional groups, including the Atg1 protein kinase complex (*AtATG101*), ATG9/2/18 transmembrane complex (*AtATG9*), ubiquitin-like ATG8-PE conjugation pathway (*AtATG3*, *AtATG8b*, *AtATG8c*, *AtATG8e*, *AtATG8f*, *AtATG8g*, and *AtNBR1*), and ATG12-ATG5 conjugation pathway (*AtATG5*, *AtATG10*, and *AtATG12a*), further demonstrated the enhancement of autophagy in transgenic plants, especially in mature leaves (Figures 6B,D,F,H,J,L,N,P,R,T,V,X). In young leaves, under LN, a decrease expression pattern between transgenic plants and control plants of four amino acid transporters (*AtAAP1*, *AtAAP5*, *AtAAP6*, and *AtLHT1*) and three N assimilation genes (*AtGLN1;1*, *AtGLU1*, and *AtNIA1*) (Figures 5A,E,G,I,K,M,O), here, we can explain this by the developmental differences between transgenic plants and WT, exactly, the young leaves of transgenic plants under LN were during the period of their vigorous growth, thus, it was a sink for whole plants at a basal autophagy level. Another distinct result was the expression level of *AtATG9* in young leaves, showing a significant decrease in transgenic plants vs. WT under NN (Figure 6O). We might conclude that *AtATG9* was at a basal expression level due to the relatively low expression level for both transgenic plants and WT.

Amino acids are the currency of N transfer in plants (Ortiz-Lopez et al., 2000). In this study, though no obvious differences were captured between transgenic plants and WT, a relative high level of amino acid-N was observed in inflorescences (Figure 3C), indicating a huge amount of N was required in both transgenic plants and control plants. The significant increase in young leaves of transgenic plants illustrated a relatively more available N (Figure 3B), which might contribute to the protein synthesis for young tissues. The increase in mature leaves under NN might derive from protein degradation (Figure 3A), which was also confirmed by the increased expression levels of amino acid transporters (*AtAAP1*, *AtAAP5*, *AtAAP6*, and *AtLHT1*) and N assimilation genes (*AtGLN1;1*, *AtGLU1*, and *AtNIA1*) in transgenic plants vs. WT (Figures 5A,E,G,I,K,M,O). Under NN, an increased expression level was observed in the roots for most of the genes responsible for N uptake (*AtAMT1;1*, *AtAMT1;2*, *AtAMT1;3*, *AtNRT2.1*, and *AtNRT2.2*) in transgenic plants (Figures 4A–C,E,F), and this might explain why the level of amino acid-N was significantly higher in transgenic plants than WT, that is, more N acquisition *via* transgenic plants roots under NN condition.

Since the plants grown hydroponically are not convenient for seeds collection, in this study, we further demonstrated the effect of *CsATG101* on *A. thaliana* reproductive growth in soil. Both in soil and in nutrient solution conditions, under LN, the transgenic plants tend to be purple (Figures 2A, 7A), indicating a higher content of anthocyanin accumulation and an improved tolerance to N deficiency, as previous study reported in apple for the overexpression of *MdATG18a* (Sun et al., 2018). At vegetative stage, the decrease in N accumulation in transgenic plants vs. WT demonstrated that the *CsATG101* reduced N uptake from soil under LN. By contrast, the increased N accumulation indicated an enhancement of N uptake in transgenic plants under NN (Figure 7E). At reproductive stage, the stubble of transgenic plants was significantly lower in biomass content and N accumulation than that in the WT under both conditions (Figures 8D,F), indicating a reduction of N in the stubble mediated by *CsATG101*. Therefore, N accumulation allocated into seeds was significantly higher in transgenic plants than WT (Figure 8H). In contrast to the results in hydroponic culture, the allocation of N accumulation in APER decreased under NN, while not differed under LN (Supplementary Figure 6H). This discrepancy was mainly attributed to the differences of harvest parts. Under hydroponic culture, we defined APER as the harvest part, thus, the elevated rosette leaves biomass reduced the biomass allocated in APER in transgenic plants. Under soil condition, since no additional N was supplied after bolting, N remobilization from earlier sources of rosettes would occur, which has been confirmed in early reports (Sun et al., 2018). Therefore, less N remained in stubble and relative more was allocated into seeds. Overall, these results illustrated that overexpression of *CsATG101* improved the growth of rosette leaves under NN condition and a relatively less residual N in stubble remained. For tea plants, the vigorously vegetative growth is desirable to achieve elevated yield of tender shoots. Here, we demonstrated that in *A. thaliana*, *CsATG101* improved the rosette leaves growth under NN. Thus, *CsATG101* was supposed to improve tender leaves growth when N is adequate. Meanwhile, tea plant is a commercial crop with round-by-round fresh leaves harvested annually, and a batch of tender shoots developed under suitable climate, making multiple sinks competed for the nutrients from soil or the sources. However, for the planation without N supply in time, the N uptake from soil is limited due to the continuous consumption, thus the mature leaves became the main sources (Zhang et al., 2020). In our study, we also illustrated that at reproductive stage, both the seed yield and stubble biomass, N concentration and accumulation were lower in transgenic plants, while a relative higher N accumulation was distributed into seeds of transgenic plants, in contrast to WT. This finding was almost in agreement with the growth regularity of tea plants, indicating a decrease in original source mature leaves, which contributed to the multiple sinks of tender shoots. These results indicated that *CsATG101* improved N utilization with different regulation patterns depend on the status of plants and in response to the environmental N conditions.

## Data Availability Statement

The original contributions presented in the study are included in the article/Supplementary Material, further inquiries can be directed to the corresponding author/s.

## Author Contributions

HZ and WH conceived and designed the study. WH and DM performed the experiments. XH, LX, and EZ participated the experiment of N determination. WH analyzed the data and prepared the manuscript. HZ revised the manuscript. PW, MW, FG, YW, and DN reviewed the manuscript. All authors have read and approved the manuscript.

## Conflict of Interest

The authors declare that the research was conducted in the absence of any commercial or financial relationships that could be construed as a potential conflict of interest.

## Publisher’s Note

All claims expressed in this article are solely those of the authors and do not necessarily represent those of their affiliated organizations, or those of the publisher, the editors and the reviewers. Any product that may be evaluated in this article, or claim that may be made by its manufacturer, is not guaranteed or endorsed by the publisher.

## Footnotes

1
http://tpia.teaplant.org/index.html

2
http://plants.ensembl.org/index.html
