# Supplementary material for: CsATG101 Delays Growth and Accelerates Senescence Response to Low Nitrogen Stress in Arabidopsis thaliana
Source: Front Plant Sci. 2022 May 10;13:880095. doi: 10.3389/fpls.2022.880095 (PMC9127664; doi:10.3389/fpls.2022.880095)
Supplement: Supplementary file 1 [file Data_Sheet_1.zip › Supplementary/Supplementary Fig.S1.docx]

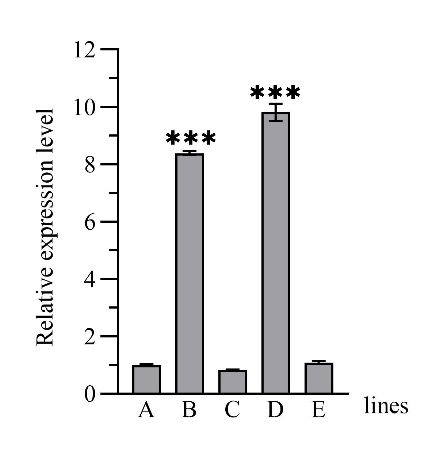


**Figure. S1** The relative expression level of *CsATG101* in five lines of *CsATG101* overexpression *Arabidopsis thaliana*. The expression level in line A was set as “1”. Data are means±SD (n≥3). ANOVA was performed and asterisks indicate significant difference from line A. ****p*＜0.001(extremely significant).
