# Supplementary material for: CsATG101 Delays Growth and Accelerates Senescence Response to Low Nitrogen Stress in Arabidopsis thaliana
Source: Front Plant Sci. 2022 May 10;13:880095. doi: 10.3389/fpls.2022.880095 (PMC9127664; doi:10.3389/fpls.2022.880095)
Supplement: Supplementary file 1 [file Data_Sheet_1.zip › Supplementary/Supplementary Fig.S2.docx]

**
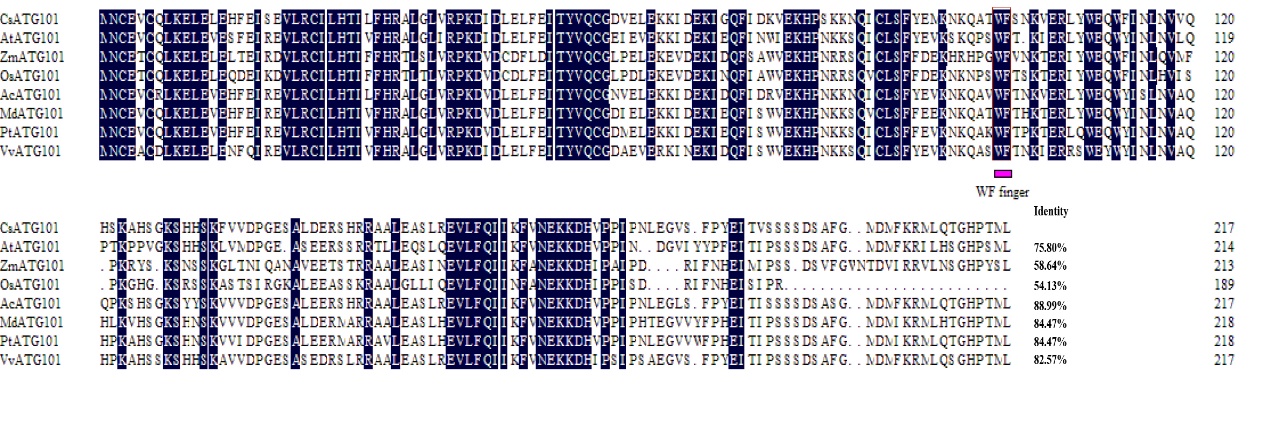
**

**Figure S2** Alignment of deduced amino acid sequences of CsATG101 with other ATG101s. AtATG101 (*Arabidopsis thaliana*, At5g66930), ZmATG101(*Zea mays*, Zm00001d051033), OsATG101(*Oryza sativa*, Os12t0446700), AcATG101 (*Actinidia chinensis*, CEY00_Acc06055), MdATG101 (*Malus domestica*, MD02G0140200), PtATG101(*Populus trichocarpa*, POPTR_007G037800v3) and VvATG101(*Vitis vinifera*, VIT_00s0253g0007). Red box indicates the conserved WF finger, and the identity at the end of the sequences represent the values of protein similarity with CsATG101. The other seven deduced amino acid sequences were downloaded from Ensembl Plants (<http://plants.ensembl.org/index.html>).
