# Supplementary material for: CsATG101 Delays Growth and Accelerates Senescence Response to Low Nitrogen Stress in Arabidopsis thaliana
Source: Front Plant Sci. 2022 May 10;13:880095. doi: 10.3389/fpls.2022.880095 (PMC9127664; doi:10.3389/fpls.2022.880095)
Supplement: Supplementary file 1 [file Data_Sheet_1.zip › Supplementary/Supplementary Fig.S3.docx]

**
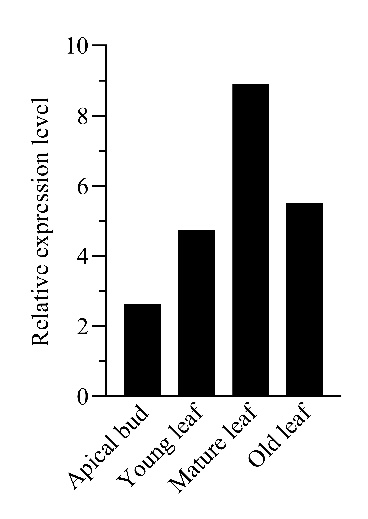
**

**Figure. S3** The expression data of *CsATG101* for the sequenced cultivar *Suchazao* downloaded from Tea Plant Information Archive (TPIA, http://tpia.teaplant.org/download.html).
