# Supplementary material for: CsATG101 Delays Growth and Accelerates Senescence Response to Low Nitrogen Stress in Arabidopsis thaliana
Source: Front Plant Sci. 2022 May 10;13:880095. doi: 10.3389/fpls.2022.880095 (PMC9127664; doi:10.3389/fpls.2022.880095)
Supplement: Supplementary file 1 [file Data_Sheet_1.zip › Supplementary/Supplementary Fig.S4.docx]

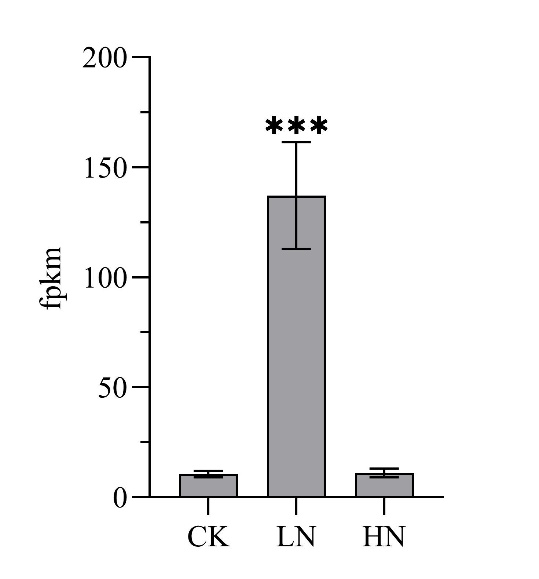


**Figure. S4 Expression levels of *CsATG101* in response to different N treatments**. The data were selected from the transcriptomic treated with different N levels published in our earlier reports (Zhang et al., 2020). CK, N-replete control; LN, Low N, 0.28 mM N; HN, High N, 2.8 mM N.

Zhang, X., Liu, H., Pilon-Smits, E., Huang, W., and Wang, P., et al. (2020). Transcriptome-wide analysis of nitrogen-regulated genes in tea plant (*Camellia sinensis* L. O. Kuntze) and characterization of amino acid transporter *CsCAT9.1*. *Plants* 9, 1218. doi:10.3390/plants9091218
