# Supplementary material for: CsATG101 Delays Growth and Accelerates Senescence Response to Low Nitrogen Stress in Arabidopsis thaliana
Source: Front Plant Sci. 2022 May 10;13:880095. doi: 10.3389/fpls.2022.880095 (PMC9127664; doi:10.3389/fpls.2022.880095)
Supplement: Supplementary file 1 [file Data_Sheet_1.zip › Supplementary/Supplementary Fig.S5.docx]

**
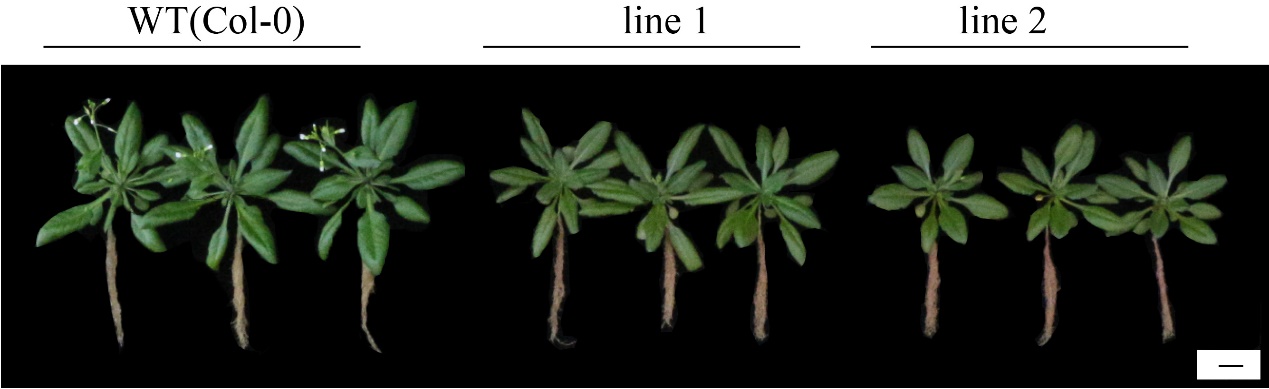
**

**Figure. S5** Growth performance of two transgenic *Arabidopsis* lines and wild type hydroponically cultured for three weeks under NN. NN, normal nitrogen, 5 mM N (2.5 mM NH_4_NO_3_). Scale bar =1 cm.
