# Supplementary material for: CsATG101 Delays Growth and Accelerates Senescence Response to Low Nitrogen Stress in Arabidopsis thaliana
Source: Front Plant Sci. 2022 May 10;13:880095. doi: 10.3389/fpls.2022.880095 (PMC9127664; doi:10.3389/fpls.2022.880095)
Supplement: Supplementary file 1 [file Data_Sheet_1.zip › Supplementary/Supplementary Fig.S6.docx]

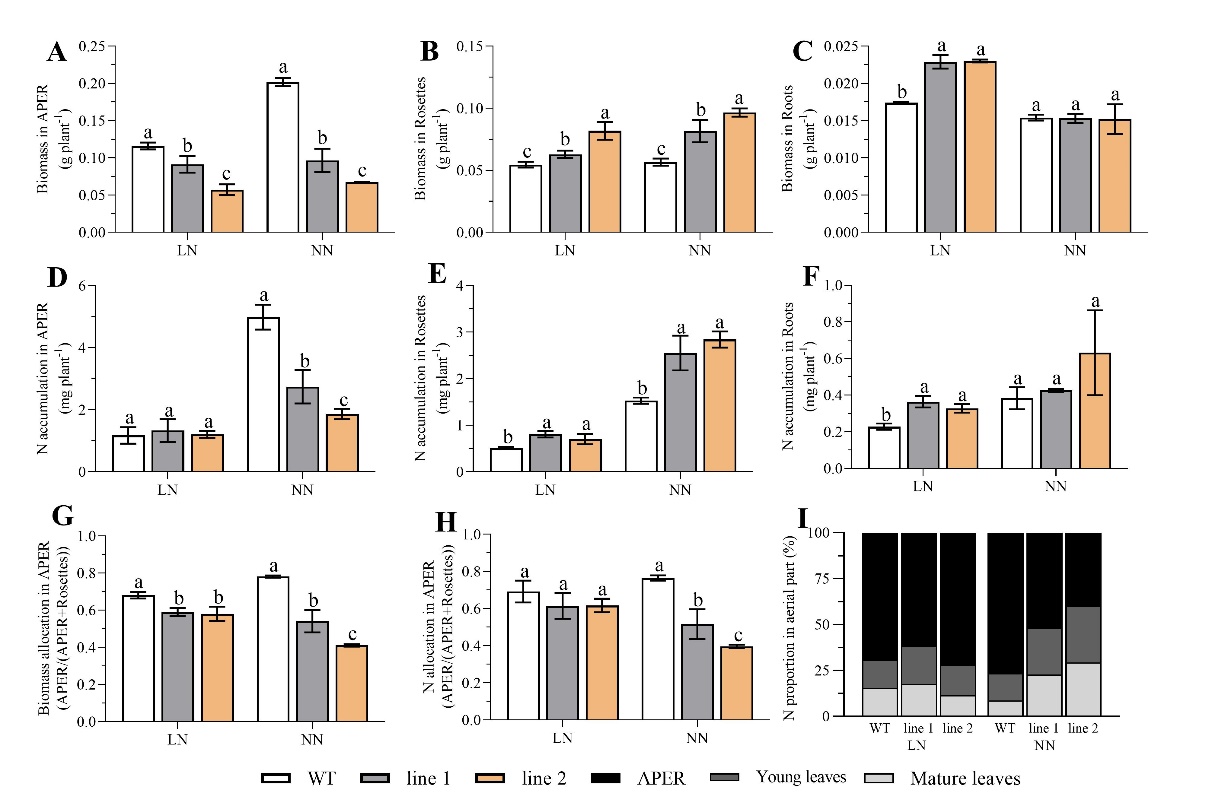


**Figure. S6** A comparison of separated biomass and N accumulation and allocation between two transgenic *Arabidopsis* lines and wild type. Plants were hydroponically cultured for three weeks under NN, and then under LN and NN for two weeks. The biomass of APER (**A**), Rosettes (**B**) and Roots (**C**) (n ≥ 5). The N accumulation in APER (**D**), Rosettes (**E**) and Roots (**F**) (n ≥ 5). **G,** The biomass allocation in APER (APER biomass/biomass of APER and Rosettes). **H,** The N allocation in APER (APER N accumulation/N accumulation of APER and Rosettes). **I**, N accumulation proportion for APER, young leaves, and mature leaves within aerial part (n ≥ 5). Three biological replicates were performed. Data are means ± SD. Significant differences are indicated by letters (ANOVA; P ≤ 0.05). WT, wild type (Col-0); line 1 and line 2, two transgenic lines; APER, Aerial part excluding rosette. LN, low nitrogen, 0.25 mM N (0.125 mM NH_4_NO_3_); NN, normal nitrogen, 5 mM N (2.5 mM NH_4_NO_3_).
